# D-dimer serves as predictor of plastic bronchitis or necrotizing pneumonia in children with *Mycoplasma pneumoniae* pneumonia

**DOI:** 10.3389/fped.2025.1604253

**Published:** 2025-07-15

**Authors:** Yanke Yue, Tiantian Lian, Lei Kang, Shuai Liu, Wenjin Geng, Meixian Xu

**Affiliations:** ^1^ Department of Intensive Care Unit, Hebei Children’s Hospital, Shijiazhuang, Hebei, China; ^2^ Department of Psychological and Behavioural Science, Hebei Children’s Hospital, Shijiazhuang, Hebei, China; ^3^ Department of Emergency, Hebei Children’s Hospital, Shijiazhuang, Hebei, China

**Keywords:** D-dimer, plastic bronchitis, necrotizing pneumonia, *mycoplasma pneumoniae* pneumonia, children

## Abstract

**Objective:**

This study aimed to explore risk factors for plastic bronchitis (PB) or necrotizing pneumonia (NP) in children with *Mycoplasma pneumoniae* pneumonia (MPP).

**Methods:**

This is a retrospective, observational cohort study, which was conducted at the Hebei Children's Hospital, Shijiazhuang, Hebei, China. This study compared the clinical characteristics between children with MPP who developed PB or NP and children with MPP who did not develop PB or NP. Variables with a *P*-value <0.1 in the univariate logistic regression analysis were further analyzed in the multivariate logistic regression analysis.

**Results:**

One hundred and seven hospitalized children with MPP were retrospectively enrolled in this study. Three (3/107, 2.80%) patients were admitted with severe pneumonia, and sixty-nine (69/107, 64.49%) patients required for non-invasive ventilation after admission. The incidence of macrolide-unresponsive *Mycoplasma pneumoniae* pneumonia (MUMPP) was 39.25% (42/107), and the incidence of refractory *Mycoplasma pneumoniae* pneumonia (RMPP) was 9.35% (10/107). Thirteen (13/107, 12.15%) patients were diagnosed with PB or NP during hospitalization. Logistic regression analysis showed that the D-dimer (DD) level [odds ratio [OR] 1.28, 95% confidence interval [CI] 1.07–1.61; *P* = 0.013] was independently and positively associated with the risk of PB or NP occurring. Receiver operating characteristic (ROC) analysis showed that the best cutoff point for D-dimer in predicting PB or NP is 2.44 (mg/L) (AUC = 0.85, 95% CI: 0.76–0.95, sensitivity: 92.31%, specificity: 75.53%, *P* < 0.001*).

**Conclusions:**

This study found that the elevated DD level (≥2.44 mg/L) has a predicting value for the progression of children with MPP to the composite outcome of PB or NP. However, due to the limited number of PB cases, its specific prediction for PB needs further verification.

## Introduction

*Mycoplasma pneumoniae* pneumonia (MPP) is one of the most common respiratory infections in children, and its incidence has been increasing year by year since 2000 (1), especially in China (2). *Mycoplasma pneumoniae* (MP) infections have increased significantly after the COVID-19 pandemic (3). The reported rate of intensive care unit (ICU) admission was 10% to 16.3% (4, 5) in those patients with MPP. Given the rising incidence of MPP, severe complications requiring ICU admission have increased proportionally (6). In recent years, with the increasing of macrolide-resistant *M. pneumoniae* (MRMP), clinicians have paid more attention to exploring risk factors of macrolide-unresponsive *Mycoplasma pneumoniae* pneumonia (MUMPP) or refractory *Mycoplasma pneumoniae* pneumonia (RMPP). MP can damage the lining of the respiratory tract, including the throat, windpipe, and lungs. Although MP infections are primarily self-limiting and mild, untreated ones may still result in a poor prognosis, especially in children.

Plastic bronchitis (PB) (7) and necrotizing pneumonia (NP) (8) are both complications of MPP, which may induce persistent hyperpyrexia, hypoxia, respiratory distress and so on. Timely bronchoscopy and bronchoalveolar lavage can achieve good therapeutic effects in children with PB and prevent the occurrence of sequelae (9). Early use of low molecular weight heparin can reduce the risk of NP (10). Good predictive markers can help identify high-risk groups for PB or NP early and receive appropriate treatment early to improve prognosis. Prognostic models for PB (7) and NP (8) have already been constructed. However, few studies reported the risk factors for both PB and NP. Luo et al. (8) found that the fever duration, bacterial co-infection, chest pain, levels of lactic dehydrogenase (LDH), C-reactive protein (CRP), and DD could predict necrotizing MPP.

Furthermore, Yang et al. (11) found that patients with MPP and concurrent PB had higher levels of neutrophil, CRP, procalcitonin (PCT), DD, LDH and aspartate aminotransferase (AST) than those without. In clinical practice, PB and NP require urgent intervention (bronchoscopy or anticoagulation). The composite endpoint design aligns more with clinical decision-making logic and avoids repeated testing of a single indicator. Then, we hypothesized if we could find common predictors for PB or NP in children with MPP.

In study, we aimed to explore the clinical characteristics of children with MPP and found risk factors that predict both PB or NP.

## Methods

### Study designs and patients

This retrospective, observational cohort study was conducted at the Hebei Children's Hospital, Shijiazhuang, Hebei, China. From November 2021 to June 2023, hospitalized children with MPP were retrospectively enrolled in our study. The inclusion criteria were as follows: (i) hospitalized children; (ii) diagnosed with MPP. The exclusion criteria were as follows: (i) with incomplete laboratory test results; (ii) absence of fever during hospitalization; (iii) with missing medical records; (iv) bronchoscopy or chest CT was not performed. All laboratory tests (such as blood routine, liver and kidney function, coagulation, etc.) were completed on the day of admission. And the children with abnormal chest imaging at discharge are scheduled for a follow-up examination 1–2 weeks to avoid early misdiagnosis as much as possible. All children underwent bronchoscopy within the first 3 days of hospitalization. Depending on the severity of the disease, some children may be admitted to the hospital for a repeat bronchoscopy within 1 month after discharge. This study was approved by the Ethics Committee of Hebei Children's Hospital (Approved number: 202136). Moreover, the informed consent was waived for the retrospective design of this study. This study did not involve human or animal experiments.

### Data collection and definitions

This study retrospectively collected characteristics (age, sex), initial symptoms of MP infection, temperature, chest imaging data and laboratory tests, diagnosis, therapy, and prognosis. More than two pediatricians collected all the data at the same time. Discrepancies in data interpretation were resolved by consensus with a senior clinician. MPP is diagnosed according to the Evidence-based Guideline for the Diagnosis and Treatment of *Mycoplasma Pneumoniae* Pneumonia in Children (2023) (12). MUMPP is defined as persistent fever in patients with MPP after ≥3 days of appropriate use of macrolide antibiotics (13). RMPP is diagnosed as persistent fever or worsening of clinical symptoms and chest imaging findings in patients with MPP after ≥ 7 days of appropriate use of macrolide antibiotics (14). Severe pneumonia is diagnosed according to Guidelines for the Management of Community-acquired Pneumonia in Children (2024) (15). The indication of non-invasive ventilation refers to the Guidelines for the Management of Community-acquired Pneumonia in Children (2024) (15). The diagnosis of PB requires a combination of clinical symptoms (such as severe coughing, dyspnea, etc.), imaging examinations (chest CT showing bronchial tube shadows), and bronchoscopic findings of plastic secretions blocking the airways (7, 16). NP is an imaging diagnosis, and the chest CT is the gold standard for diagnosing NP, which is based on pulmonary consolidation and manifests as multiple low-density thin-walled cavities (8).

### Statistical analysis

Continuous variables were expressed as medians (quartiles), and categorical variables were described as frequencies (%). The enrolled children with MPP were divided into a poor prognosis (with PB or NP) group and a non-poor prognosis group. Continuous variables were compared using the Student's t test or Mann–Whitney *U*-test, while categorical data were compared using the chi-square test or Fisher's exact test. All variables with *P* value <0.1 in the univariate logistic regression analysis were further analyzed in the multivariate logistic regression analysis. Receiver operating characteristic (ROC) analysis and Kaplan–Meier survival analysis were performed to assess the variables to predict poor prognosis (with PB or NP). Moreover, the predictive effectiveness was evaluate basing on the area under the curve (AUC), and an AUC >0.9 indicated high predictive performance (13). All the statistical analyses were conducted in R software (version 4.3.0). A *P* value ≤ 0.05 was considered to indicate statistical significance.

## Results

### Study population

**Figure 1:**
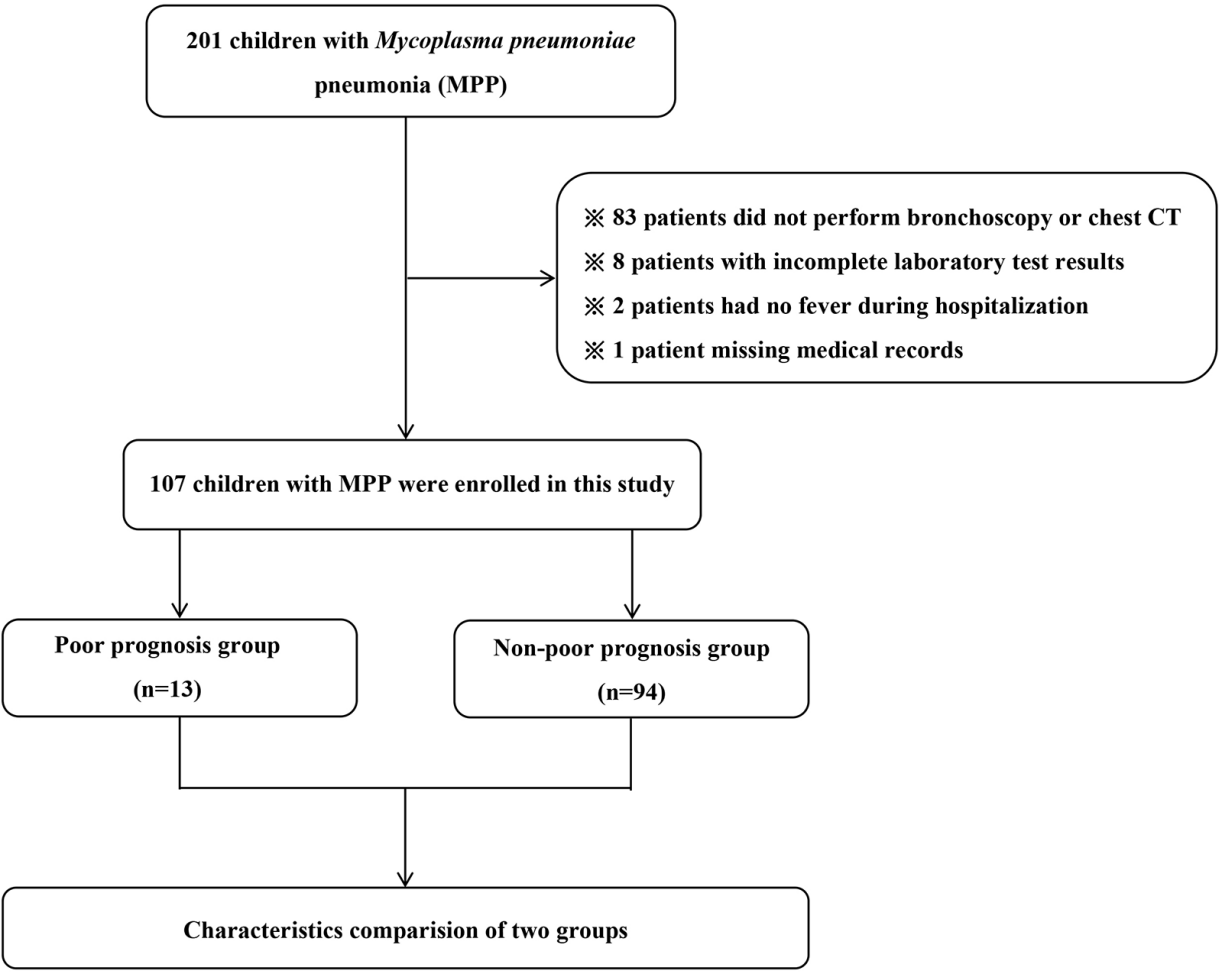
The patient inclusion flow chart.

From November 2021 to June 2023, there were 201 children with MPP hospitalized at Hebei Children's Hospital. Then, ninety-four patients were excluded (83 patients did not perform bronchoscopy or chest CT, 8 patients with incomplete laboratory test results, 2 patients had no fever during hospitalization and 1 patient with missing medical records). At last, there were 107 children with MPP were enrolled in our study. Details are presented in Figure 1.

### Clinical characteristics of 107 children with MPP

The median age was 7.00 [Interquartile range (IQR) 5.00–9.00] years, and more than 50% (51.40%, 55/107) of all patients were male. About half (48.60%, 52/107) of children with MPP started with fever and cough. The median duration of fever before azithromycin use was 7.00 (IQR 4.00–10.00) days. Three (3/107, 2.80%) patients were admitted with severe pneumonia, and sixty-nine (69/107, 64.49%) patients needed non-invasive ventilation after admission. And two (2/107, 1.87%) patients were admitted to the intensive care unit (ICU). More than half of patients received glucocorticoid therapy or non-macrolide antibiotics (68.22% and 55.14%, respectively). Sixty-two (62/107, 57.94%) patients received tetracycline/quinolone therapy. The median length of hospitalization was 9.00 (IQR 7.00–13.00) days. The incidence of macrolide-unresponsive *Mycoplasma pneumoniae* pneumonia (MUMPP) was 39.25% (42/107), and the incidence of refractory *Mycoplasma pneumoniae* pneumonia (RMPP) was 9.35% (10/107). Thirteen (13/107, 6.84%) patients were diagnosed with PB or NP during hospitalization (Ten cases with NP, seven with PB, and four with both PB and NP). The re-admission rate within one month was 7.48% (8/107). All results are shown in Table 1.

**Table 1:** Clinical characteristics of 107 children with *Mycoplasma pneumoniae* pneumonia (MPP).

Characteristics	Total (*n* = 107)
Demographic data	
Age (years) (median, IQR)	7.00 (5.00–9.00)
Sex (male) (*n*, %)	55 (51.40%)
Initial symptoms	
Fever and cough (*n*, %)	52 (48.60%)
Fever (*n*, %)	35 (32.71%)
Cough (*n*, %)	20 (18.69%)
Wheezing (*n*, %)	4 (3.74%)
Laboratory tests	
Inflammatory indicators	
C-reactive protein (CRP) (mg/L) (median, IQR)	19.15 (3.79–44.28)
Procalcitonin (PCT) (ng/ml) (median, IQR)	0.10 (0.05–0.21)
Blood routine examination	
White blood cell (WBC) (*10^−9^/L) (median, IQR)	8.60 (7.10–11.80)
Neutrophil (*10^−9^/L) (median, IQR)	5.48 (4.30–7.81)
Hemoglobin (Hb) (g/L) (median, IQR)	125.00 (115.45–131.00)
Liver function	
Lactic dehydrogenase (LDH) (U/L) (median, IQR)	305.00 (250.50–404.50)
Alanine aminotransferase (ALT) (U/L) (median, IQR)	15.00 (10.00–26.50)
Aspartate aminotransferase (AST) (U/L) (median, IQR)	24.00 (19.00–33.00)
Coagulation function	
D-dimer (DD) (mg/L) (median, IQR)	1.19 (0.43–3.07)
Fibrinogen (Fib) (mg/dl) (median, IQR)	3.82 (3.29–4.65)
Co-infections	
Co-bacterial infection (*n*, %)	52 (48.60%)
Co-viral infection (*n*, %)	26 (24.30%)
Therapy	
Glucocorticoid (*n*, %)	73 (68.22%)
Tetracycline/quinolone (*n*, %)	62 (57.94%)
Non-macrolide antibiotics (*n*, %)	59 (55.14%)
Immunoglobulin (*n*, %)	7 (6.54%)
Admitted with severe pneumonia (*n*, %)	3 (2.80%)
The highest body temperature during the course (℃) (median, IQR)	39.1 (38.6–40.0)
Duration of fever before azithromycin use (days) (median, IQR)	7.00 (4.00–10.00)
Duration of cough before azithromycin use (days) (median, IQR)	7.00 (4.00–11.00)
Need for non-invasive ventilation (*n*, %)	69 (64.49%)
Intensive care unit (ICU) admission (*n*, %)	2 (1.87%)
Length of hospitalization (days) (median, IQR)	9.00 (7.00–13.00)
Macrolide-unresponsive MPP (MUMPP) (*n*, %)	42 (39.25%)
Refractory MPP (RMPP) (*n*, %)	10 (9.35%)
Re-admission within 1 month for MPP (*n*, %)	8 (7.48%)
Poor prognosis (*n*, %)	13 (12.15%)
Necrotizing pneumonia (NP) (*n*, %)	10 (9.35%)
Plastic bronchitis (PB) (*n*, %)	7 (6.54%)

### Comparisons of the clinical characteristics between the poor prognosis (progress to PB or NP) and non-poor prognosis groups of 107 children with MPP

Patients in the poor prognosis group (progress to PB or NP) had much higher levels of alanine aminotransferase (ALT), DD, and lactic dehydrogenase (LDH) than those patients without poor prognosis. (*P* < 0.05). There were significantly more patients with PB or NP coinfected with bacteria than those without (84.62% vs. 43.62%, *P* = 0.013). Moreover, patients in the poor prognosis group (progress to PB or NP) had significantly more extended hospital stays than those without poor prognosis [15.00 (IQR 9.00–18.00) days vs. 9.00 (IQR 7.00–12.00) days, *P* = 0.033]. In addition, the poor prognosis group also had a higher rate of re-admission within 1 month for MPP and a higher incidence of RMPP than the non-poor prognosis group (*P* < 0.05). The demographic data (age and sex), initial symptoms of MPP, the highest body temperature during the course, duration of cough before azithromycin, and proportions of ICU admission were with no significant differences between the poor prognosis (progress to PB or NP) and the non-poor prognosis group. The details of comparisons of the two groups are shown in Table 2.

**Table 2:** Comparisons of clinical characteristics between poor prognosis (progress to PB or NP) and non-poor prognosis groups in 107 children with MPP.

Characteristics	Non-poor prognosis (*n* = 94)	Poor prognosis (*n* = 13)	*P*-value
Demographic data			
Age (years) (median, IQR)	7.00 (5.00–9.00)	7.00 (5.00–7.00)	0.499
Sex (male) (*n*, %)	45 (47.87%)	10 (76.92%)	0.095
Initial symptoms			
Fever and cough (*n*, %)	46 (48.94%)	6 (46.15%)	1.000
Fever (*n*, %)	30 (31.91%)	5 (38.46%)	0.754
Cough (*n*, %)	18 (19.15%)	2 (15.38%)	1.000
Wheezing (*n*, %)	4 (4.26%)	0 (0.00%)	1.000
Laboratory tests			
Inflammatory indicators			
CRP (mg/L) (median, IQR)	18.84 (3.73–43.24)	21.00 (10.08–48.89)	0.678
PCT (ng/ml) (median, IQR)	0.09 (0.05–0.19)	0.21 (0.06–0.32)	0.167
Blood routine examination			
WBC (*10^−9^/L) (median, IQR)	8.55 (7.03–11.15)	9.60 (8.10–14.50)	0.228
Neutrophil (*10^−9^/L) (median, IQR)	5.28 (4.22–7.37)	6.79 (5.46–11.39)	0.079
Hemoglobin (Hb) (g/L) (median, IQR)	126.00 (116.00–131.00)	120.00 (113.00–128.00)	0.159
Liver function			
LDH (U/L) (median, IQR)	303.50 (246.00–377.50)	531.00 (401.00–548.00)	0.001^*^
ALT (U/L) (median, IQR)	13.00 (10.00–24.00)	23.00 (18.00–61.00)	0.007^*^
AST (U/L) (median, IQR)	23.00 (19.00–31.00)	30.00 (23.00–37.00)	0.076
Coagulation function			
DD (mg/L) (median, IQR)	1.09 (0.35–2.41)	4.07 (2.61–8.21)	<0.001^*^
Fib (mg/dl) (median, IQR)	3.74 (3.25–4.49)	4.65 (3.74–5.02)	0.085
Co-infections			
Co-bacterial infection (*n*, %)	41 (43.62%)	11 (84.62%)	0.013^*^
Co-viral infection (*n*, %)	21 (22.34%)	5 (38.46%)	0.298
Therapy			
Glucocorticoid (*n*, %)	61 (64.89%)	12 (92.31%)	0.058
Tetracycline/quinolone (*n*, %)	51 (54.26%)	11 (84.62%)	0.075
Non-macrolide antibiotics (*n*, %)	49 (52.13%)	10 (76.92%)	0.165
Immunoglobulin (*n*, %)	5 (5.32%)	2 (15.38%)	0.202
Admitted with severe pneumonia (*n*, %)	1 (1.06%)	2 (15.38%)	0.008^*^
The highest body temperature during the course (℃) (median, IQR)	39.0 (38.5–39.8)	40.0 (39.0–40.1)	0.148
Duration of fever before azithromycin use (days) (median, IQR)	7.00 (4.00–10.00)	9.00 (8.00–10.00)	0.074
Duration of cough before azithromycin use (days) (median, IQR)	7.00 (4.00–11.00)	9.00 (8.00–10.00)	0.125
Need for non-invasive ventilation (*n*, %)	58 (61.70%)	11 (84.62%)	0.131
ICU admission (*n*, %)	2 (2.13%)	0 (0.00%)	1.000
Length of hospitalization (days) (median, IQR)	9.00 (7.00–12.00)	15.00 (9.00–18.00)	0.033^*^
Macrolide-unresponsive MPP (MUMPP) (*n*, %)	34 (36.17%)	8 (61.54%)	0.146
Refractory MPP (RMPP) (*n*, %)	4 (4.26%)	6 (46.15%)	<0.001^*^
Re-admission within 1 month for MPP (*n*, %)	5 (5.32%)	3 (23.08%)	0.055

* With statistical significance, *P* < 0.05.

### Risk factors for PB or NP in 107 children with MPP

In univariate logistic regression analysis, sex, admitted with severe pneumonia, co-bacterial infection, MUMPP, levels of DD, ALT, and LDH were associated with the poor prognosis (with PB or NP). (*P* < 0.1). Then, the multivariate regression analysis showed that a higher level of D-dimer was independently associated with the risk of PB or NP [odds ratio [OR] 1.28, 95% confidence interval [CI] 1.07–1.61, *P* = 0.013]. The results of the logistic analysis are shown in Table 3.

**Table 3:** Logistic regression analysis of risk factors of PB or NP among 107 children with MPP.

Variables	Univariate analysis			Multivariate analysis		
	OR	95% CI	*P*	OR	95% CI	*P*
DD (mg/L)	1.38	1.17–1.68	<0.001^*^	1.28	1.07–1.61	0.013^*^
Admitted with severe pneumonia	16.91	1.50–381.97	0.025^*^			
Co-bacterial infection	7.11	1.78–47.60	0.014^*^			
Sex (Male)	3.63	1.03–16.95	0.062			
MUMPP	2.82	0.87–10.00	0.088			
ALT (U/L)	1.02	1.01–1.03	0.004^*^			
LDH (U/L)	1.005	1.002–1.009	<0.001^*^			

* With statistical significance, *P* < 0.05.

### Predictive value of poor prognosis by DD in children with MPP

**Figure 2:**
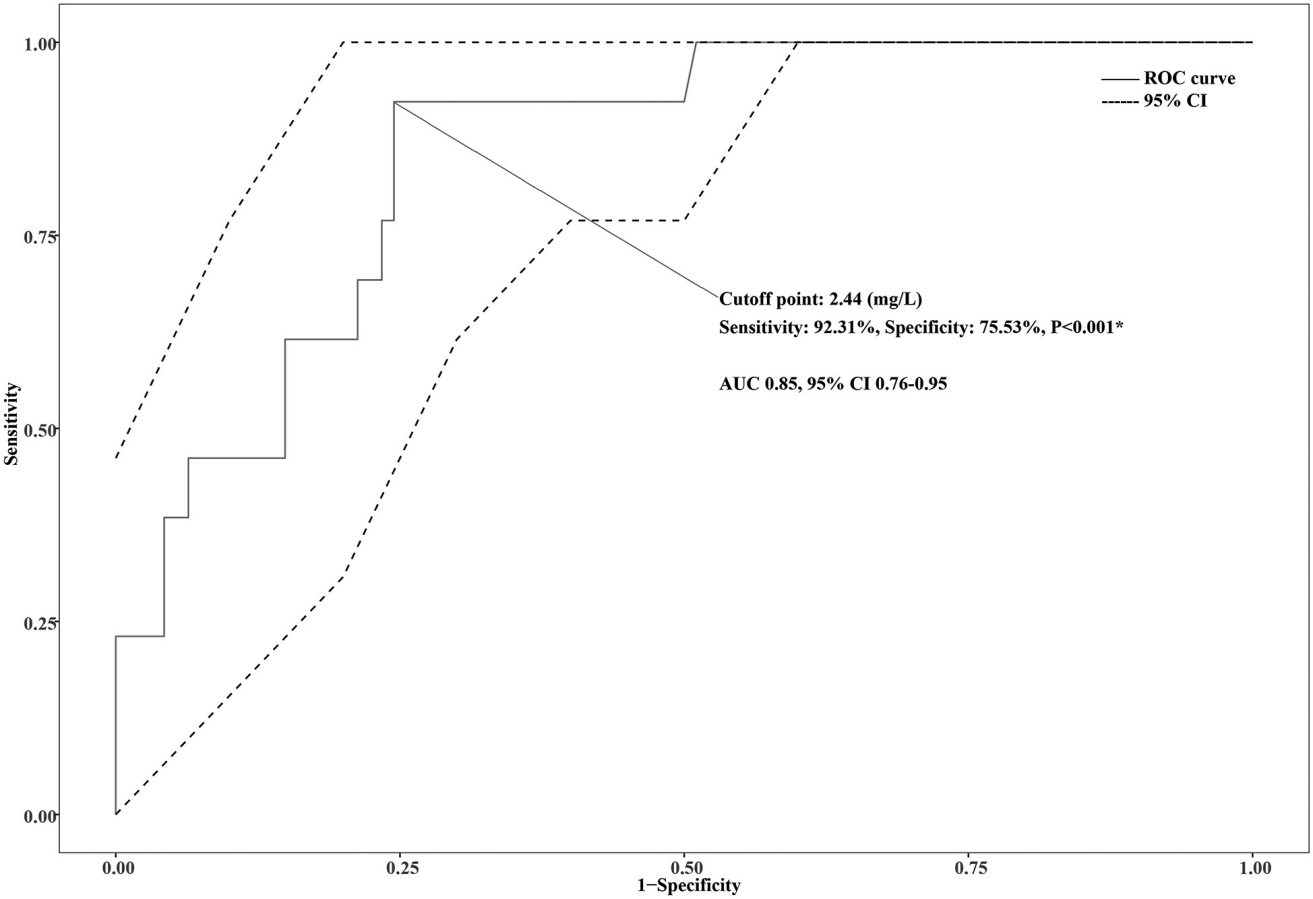
ROC curve for D-dimer (DD) to predict plastic bronchitis (PB) or necrotizing pneumonia (NP) in children with *Mycoplasma pneumoniae* pneumonia (MPP).

**Figure 3:**
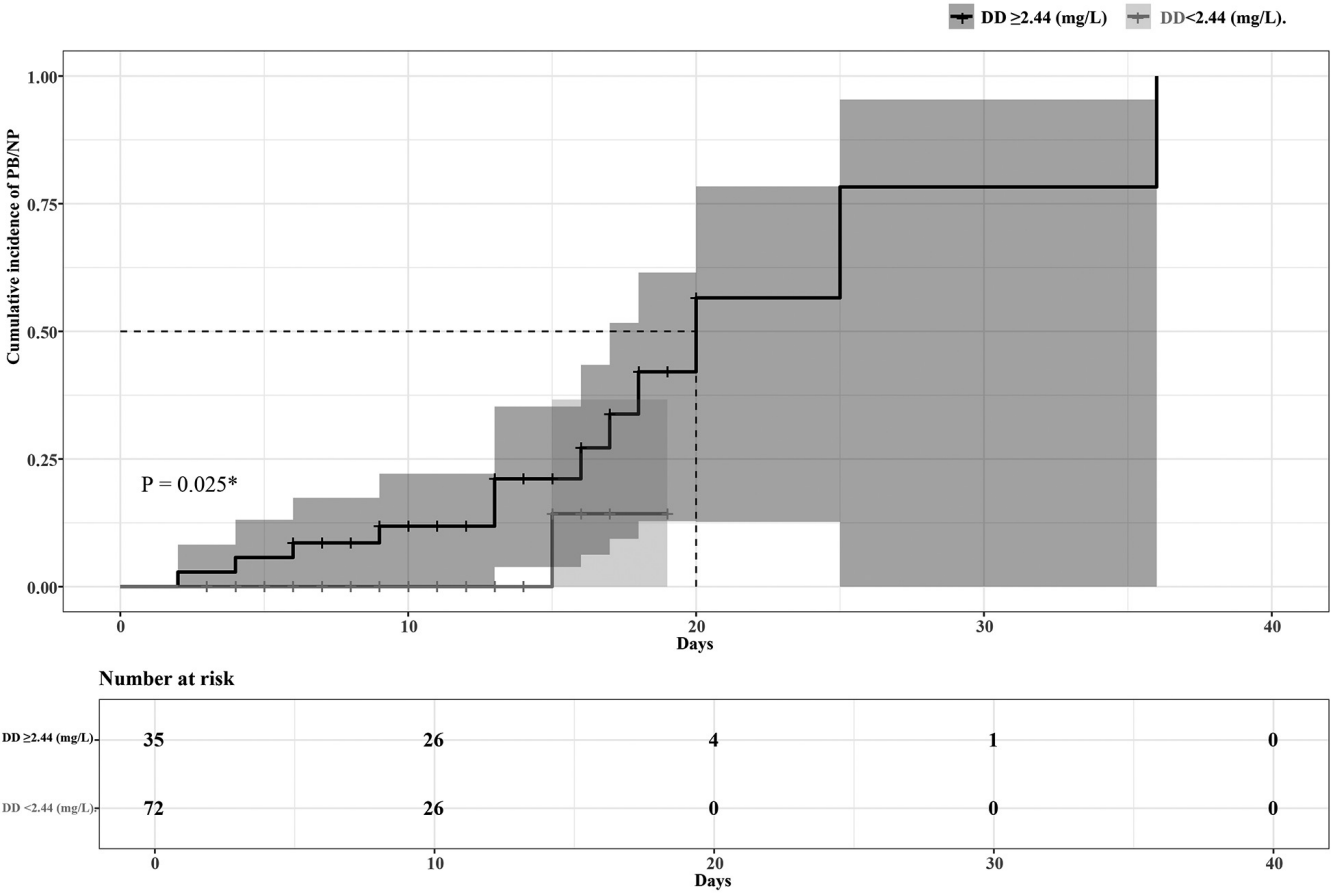
The comparison of DD according to poor prognosis (progress to PB or NP).

The ROC curve analysis showed high predictive efficacy of poor prognosis (progress to PB or NP) in children with MPP (AUC 0.85, 95% CI 0.76–0.95, *P* < 0.001). The optimal cutoff point of DD to predict poor prognosis was 2.44 mg/L, with 92.31% sensitivity and 75.53% specificity. Details are presented in Figure 2. Patients were then divided into two groups based on the optimal cutoff point of DD, and the Kaplan–Meier survival curve was plotted. As the hospital stays lengthen, patients with a DD level ≥2.44 (mg/L) had a remarkably higher risk of poor prognosis than those with a DD level <2.44 (mg/L). (*P* = 0.025). Details are seen in Figure 3.

## Discussion

Pneumonia is the most common respiratory disease in children, and the data for 2022 show that the global incidence of pneumonia exceeds 14‰ (15). MP is one of the most common pathogens that cause community-acquired pneumonia in children (13), especially for those older than 5 years old (1). Most infections with MP are mild or self-limiting, while severe cases can be life-threatening (17). PB (7) and NP (8) are both complications of MPP, which may result from lung injury. The levels of D-dimer might be positively associated with the risk of lung injury (18, 19). Li et al. (20) stated that D-dimer levels were positively correlated with the severity of MPP. Dynamic monitoring of D-dimer levels may be significant for the prognosis of children with MPP. The study found that DD level on admission was independently associated with the risk of poor prognosis (progress to PB or NP) in children with MPP (OR 1.28, 95% CI: 1.07–1.61, *P* = 0.013). Possible explanations for the predicting PB or NP risk by D-dimer level are as follows.

Community-acquired respiratory distress syndrome (CARDS) toxin was first reported by Kannan et al. (21) in 2005 and has gradually been recognized as one of the important pathogenic factors of MP (22). This toxin can directly cause cell damage as a cytotoxin and also plays an important role in the adhesion of MP and proteins associated with alveolar surfactants (22). Medina et al. (18) found that the synthesis and distribution of CARDS toxin were closely related to lung injury, and the severity of disease in naive animals was related to the level of CARDS toxin. Li et al. (23) found that CARDS toxin was positively correlated with TNF-α level in MPP cases, and they also found that CARDS toxin-induced RAW264.7 macrophages to secrete TNF-α. Shorr et al. (24) found that the coagulation system was activated in critically ill patients, and DD levels correlated with activating a cascade of pro-inflammatory cytokines.

Furthermore, TNF-α was demonstrated to be independently related to positive DD levels in critical patients (25), which might be due to its regulation function for DD (25). Then, MP could stimulate the increase of TNF levels by secreting CARDS toxin and regulate DD levels by TNF levels. Afterward, higher DD levels indicated higher levels of CARDS toxin, which causes more serious lung injury.

We agree with the literature base that DD can predict single complications (PB or NP). Li et al. (20) and Zheng et al. (10) stated that DD served as an independent risk factor of NP in children with MPP. Yang et al. (11) found that patients with MPP and concurrent PB had higher levels of DD than those with not. However, we have not found any literature confirming that DD is an independent risk factor for predicting PB. Our study is the first to find that DD is an independent predictor of the composite outcome of PB or NP, and the predictive power comes from their exact core pathogenesis (hypercoagulable state and inflammatory injury) (26). In clinical practice, PB and NP require urgent intervention (bronchoscopy or anticoagulation) (9, 10). The composite endpoint design aligns more with clinical decision-making logic and avoids repeated testing of a single indicator. However, the specific clinical manifestation classification (Isolated PB, isolated NP, or concurrent PB and NP) may depend on the difference in the interaction between the host immune response intensity and the pathogen virulence factors. Although DD ≥2.44 mg/L has a warning value for the composite outcome of PB or NP (sensitivity 92.31%), its specificity of 75% indicates a false positive risk (about 25% of children who have not progressed have DD levels exceeding this threshold). If this is used as the only basis for anticoagulation therapy or bronchoscopy, it may lead to excessive intervention and related complications. Therefore, clinical decision-making should be based on comprehensive imaging, inflammatory indicators, and dynamic monitoring rather than solely on DD thresholds. In the future, multi-parameter models must be developed to optimize risk stratification. This study used a composite endpoint (PB or NP) to improve statistical power. However, its limitations must be acknowledged: the incidence of NP (10 cases) was higher than that of PB alone (3 cases), and previous studies have suggested that DD is more strongly associated with NP (11, 20). Because the sample size of the PB subgroup was too small (*n* = 3), the predictive value of DD for PB could not be independently verified. The conclusion of the composite endpoint should be understood as the warning effect of DD on the overall risk of PB or NP, especially for identifying children with NP or mixed lesions. A larger sample size is needed to verify the specific association between DD and PB.

Previous studies have shown that MP can activate the coagulation system (27). Infection-related hypercoagulable state is the basis for the increase in DD in children with MPP. When the DD level was greater than 2.44 mg/L, the risk of developing NP or PB reached the greatest, with moderate predictive power (AUC 0.85, 95% CI: 0.76–0.95, *P* < 0.001). However, further efforts are needed to distinguish the specific effects of infection from PB or NP. Jin et al. (28) found that elevated D-dimer may be closely related to thrombosis. However, none of the children included in this study had clinically or radiologically confirmed thrombotic events (such as deep vein thrombosis, pulmonary embolism, etc.), and all cases did not have underlying diseases known to predispose to thrombosis (such as severe congenital heart disease, hematological diseases). Therefore, the observed increase in DD is more likely to reflect the local pulmonary hypercoagulability induced by MP infection rather than a secondary manifestation of extrapulmonary thrombosis. Future studies will include thrombosis screening to verify this association further. Although this study found no significant difference in hemoglobin (Hb) concentration between the two groups, it should be acknowledged that the hemolysis process may directly increase DD levels by releasing procoagulants, independent of the infection itself (29). Since this study did not systematically detect hemolysis markers such as free hemoglobin and red blood cell fragility, the potential impact of hemolysis on DD results cannot be completely ruled out. Future studies need to include more comprehensive coagulation-hemolysis indicators to verify the specific warning value of DD for PB or NP.

The incidence of MRMP in Asia has been increasing year by year since 2000 (14), and the isolation rate of MRMP in Asia was even as high as 90%–100% (13). More than 90% of MRMP infections are caused by the A2063/2064G gene mutation (30–32). The MRMP infection was generally considered a significant factor in the poor prognosis of MPP. However, in our study, MUMPP was not an independent risk factor of poor prognosis (progress to PB or NP) in patients with MPP. The explanations were as follows. First, Cheng et al. (13) found that azithromycin was still effective in children with MPP with the A2063/2064G mutation. Second, tetracycline/quinolone and glucocorticoid therapy might improve the prognosis of MUMPP. In this study, more than half of the patients received tetracycline/quinolone or glucocorticoid therapy (57.94%, 62/107; 68.22%, 73/107; respectively). This might mitigate the effect of MUMPP on prognosis. Last, all patients experienced bronchoalveolar lavage therapy during hospitalization, which may improve the prognosis in patients with MPP (33).

There are some limitations in this study. First, this study was a single-center retrospective analysis with a limited sample size, which may have affected the statistical validity and extrapolation of the results due to missing data, selection bias, and the single population/diagnosis and treatment model. Second, the retrospective design of this study resulted in a lack of long-term follow-up data. Although all children with PB or NP were clinically cured at discharge, the risk of long-term sequelae could not be assessed. Third, this study was a retrospective analysis, and the specific cellular components of PB casts were not routinely detected in clinical records, so we could not trace and obtain this information for classification analysis. We agree that understanding the composition of casts is valuable for elucidating mechanisms, and this will be a focus of future prospective studies. Last, bronchoalveolar lavage sampling is random, especially for the distal airway of patients with wedge-shaped bronchiectasis. Failure to detect casts cannot completely rule out the possibility of PB (false negative risk). We emphasized that negative bronchoalveolar lavage results must be combined with comprehensive clinical judgment.

## Conclusions

In this study, we explored the risk factors of poor prognosis (progress to PB or NP), and this can help us identify high-risk groups for PB or NP early and receive appropriate treatment early to improve prognosis. Elevated DD level (≥2.44 mg/L) has a predicting value for the progression of children with MPP to the composite outcome of PB or NP. However, due to the limited number of PB cases, its specific prediction for PB needs further verification.

## Data Availability

The data analyzed in this study is subject to the following licenses/restrictions: All data in this study can be reasonably requested from the corresponding author. Requests to access these datasets should be directed to Meixian Xu, 13833185617@163.com.
